# ITGB4 Deficiency in Airway Epithelium Aggravates RSV Infection and Increases HDM Sensitivity

**DOI:** 10.3389/fimmu.2022.912095

**Published:** 2022-07-25

**Authors:** Xizi Du, Lin Yuan, Ye Yao, Yu Yang, Kai Zhou, Xinyu Wu, Leyuan Wang, Ling Qin, Wenkai Li, Yang Xiang, Xiangping Qu, Huijun Liu, Xiaoqun Qin, Ming Yang, Chi Liu

**Affiliations:** ^1^ Department of Respiratory Medicine, National Clinical Research Center for Respiratory Diseases, Xiangya Hospital, Central South University, Changsha, China; ^2^ Department of Physiology, School of Basic Medicine Science, Central South University, Changsha, China; ^3^ School of Biomedical Sciences and Pharmacy, College of Health, Medicine and Wellbeing, University of Newcastle, Callaghan, NSW, Australia; ^4^ Priority Research Centre for Healthy Lungs, Hunter Medical Research Institute (HMRI), University of Newcastle, New Lambton Heights, NSW, Australia; ^5^ Research Center of China-Africa Infectious Diseases, Xiangya School of Medicine Central South University, Changsha, China

**Keywords:** respiratory syncytial virus, asthma, ITGB4, airway epithelial cell, infection

## Abstract

**Background:**

The heterogeneity of RSV-infected pathology phenotype in early life is strongly associate with increased susceptibility of asthma in later life. However, the inner mechanism of this heterogeneity is still obscure. ITGB4 is a down-regulated adhesion molecular in the airway epithelia of asthma patients which may participate in the regulation of RSV infection related intracellular pathways.

**Object:**

This study was designed to observe the involvement of ITGB4 in the process of RSV infection and the effect of ITGB4 deficiency on anti-RSV responses of airway epithelia.

**Results:**

RSV infection caused a transient decrease of ITGB4 expression both *in vitro* and *in vivo*. Besides, ITGB4 deficiency induced not only exacerbated RSV infection, but also enhanced HDM sensitivity in later life. Moreover, IFN III (IFN-λ) was significantly suppressed during RSV infection in ITGB4 deficient airway epithelial cells. Furthermore, the suppression of IFN-λ were regulated by IRF-1 through the phosphorylation of EGFR in airway epithelial cells after RSV infection.

**Conclusion:**

These results demonstrated the involvement of ITGB4 deficiency in the development of enhance RSV infection in early life and the increased HDM sensitivity in later life by down-regulation of IFN-λ through EGFR/IRF-1 pathway in airway epithelial cells.

## 1 Introduction

Asthma is a common chronic respiratory disease and Global initiative against asthma (GINA) 2021 strategy pinpoints that asthma is the most common chronic disease in childhood (1). Epidemiological data on asthma demonstrate that respiratory viral infections in early life are closely related to recurrent wheezing during childhood. Accumulating studies further find that the lower respiratory tract infection (LRTI) caused by respiratory syncytial virus (RSV) is the leading reason to childhood hospitalization (2, 3), which is also proposed as an important risk for asthma susceptibility (4). It is worth noting that there is significant heterogeneity of RSV infection and this variation in RSV-infected disease phenotype is strongly associate with subsequent morbidity of airway diseases. Although spontaneous resolution is the outcome for most RSV-infected individuals, part of RSV-infected patients cannot be controlled and continue to aggravate, which always have high-risk of asthma in later life (5–9). However, the mechanism regulating the discrepancy of underlying pathology processes after RSV infection is still obscure.

Airway epithelial cells (AECs) are the first cell barrier between respiratory tract and external environment that plays a critical role to initiate innate immune response after RSV infection (10). Dysfunction of bronchial epithelia even has the equal position to IgE in the pathogenesis of asthma (10). Moreover, airway epithelial cells are also the key target cells for RSV replication. Upon RSV infection, viral surface proteins firstly interact with the cell membrane of airway epithelial cells to assist the entry of RSV. Then, viral genes are transcribed by the viral polymerase in the cytoplasm, and the new viral particles are released from cytoplasm which further diffused to surrounding cells (11). Thus, breaking the limit of airway epithelial is the key event that leads to aggravation of RSV infection.

Integrins, a type of structural adhesion molecules, play important roles in structural compilation, tissue repair and homeostasis of airway epithelial cells. Of note, integrin β4 (ITGB4) is down-regulated in the airway epithelia of asthma patients which is implicated in asthma susceptibility (12). The intracellular fragment of ITGB4 contains two pairs of fibronectin-like domains, which may participate in complex intracellular pathways including RSV infection, wound repair and immune response, etc. (13–15). However, little is known about the influence of ITGB4 deficiency on RSV infection and subsequent asthma susceptibility. Therefore, we thought to further explored the influence of ITGB4 deficiency on RSV-infected airway epithelia and subsequent asthma susceptibility. First, the *in vitro* RSV-treated airway epithelial cells model and the *in vivo* RSV-infected mice model were constructed respectively to detect the differential expression of ITGB4 during RSV infection. Then, the effect and underpinning molecular mechanisms of ITGB4 deficiency on RSV infection was investigated using our well-established conditional ITGB4 knockout mice. Finally, the impact of the early enhanced RSV infection on later HDM-induced pathological changes were investigated to further verify the influence of ITGB4 deficiency on antigen sensitivity after early RSV infection.

## 2 Methods

### 2.1 Nasopharyngeal Samples

Samples were collected from children presenting to hospital with acute respiratory infection and healthy individuals visiting medical center at Hunan Provincial People’s Hospital between September 2020 and February 2021. All participants provided written informed consent, approved by the No.2020KT-52 Central South University Research Ethics Committee. Eligible patients (RSV patients’ group) were aged under 5 and diagnosed with bronchiolitis and RSV - RNA positive. The staff firmly brushed the swab along the throat surface 7 to 8 times each. After swabbing, the head of the swab was snapped off and put into a tube containing Viral Transport Medium (VTM) (16, 17). Then, nasopharyngeal samples were stored at -80°C before detection.

### 2.2 Cell Culture

Normal primary human bronchial epithelial cells (referred as HBE cells in the following text) derived from adults without clear diagnosis of lung disease, which were purchased from Lifeline Cell Technology (Frederick, MD, USA. HBE cells were cultivated as described previously (18). The cells were maintained in a Ham’s F12/DMEM (1:1) with the addition of transferrin (5 mg/mL), insulin (5 mg/mL), cholera toxin (10 ng/mL), epidermal growth factor (10 ng/mL), dexamethasone (0.1 mmol/L), bovine hypothalamus extract (15 mg/mL), BSA (0.5 mg/mL) and all-transretinoic acid (30 nmol/L), at 5% CO_2_, 37°C.

### 2.3 RSV Infection

As previous literatures described (19, 20), HBE cells were inoculated with RSV-A at1, 3, 5 multiplicity of infection (MOI), respectively. In brief, the virus suspension was quantified by plaque assay. Confluent monolayer cultures of HBE cells were plated in 6-well culture plates, and incubated with 200 ul virus suspension for 2h with gentle shaking every 15 min. The volume of virus stock was calculated according to: MOI=(pfu/ml) × (the volume of virus liquid)/number of cells. Then, virus suspension removed by a gentle wash with culture medium, followed by addition of maintenance media. RSV-infected HBE cells were continuously cultured at 37°C with 5% CO_2_.

### 2.4 SiRNA Transfection and Inhibitor Treatment

The effective ITGB4 siRNA (5’-CAGAAGAUGUGGAUGAGUU-3’) and nonsense siRNA (5’-UUCUCCGAACGUGUCACGU-3’) were synthesized and transfected into HBE cells according to our previous publications (21). In brief, when HBE cells are 40% confluent in 12-well cell culture plate, add transfection mixture (2.5 μl siRNA and 6 μl lip3000 are mixed in 60μl serum-free medium per well) and incubate for 48h. EGFR inhibitor AG1478 (Selleck Chemicals, USA) were co-cultured with HBE cells for 24 hours (10μM) to inhibit EGFR phosphorylation.

### 2.5 Quantitative RT-PCR

RNA extraction, RT-PCR and quantitative RT-PCR were performed as previously described (22). Total RNA was extracted by Trizol and quantified on a Varioskan microplate reader (Thermo Scientific, USA). cDNA preparation: Take 1ug RNA sample and operate according to the reverse transcription kit instructions (PrimeScript RT Master Mix Kit, Takara, Japan); Primer sequences are designed by PrimerBank and synthesized by Shanghai Shenggong Company. The primer sequences used are shown in **Supplementary Table 1**; qPCR procedure was following the instructions of the qPCR kit (TB Green Fast qPCR Mix Kit, Takara, Japan).

### 2.6 Western Blot

Fifty micrograms (50ug) of cell protein were extracted from HBE cells according to previous procedures (23). In brief, isolated protein from cells or lung tissues were separated by 10% SDS-PAGE and transferred to a polyvinylidene fluoride (PVDF) membrane. Then, the PVDF membrane was incubated bated with primary antibody for 12 hours and next incubated with Horseradish Peroxidase (HRP) conjugated secondary antibody. The following antibodies were used to determine the levels of ITGB4 (ab197772, Abcam, USA), EGFR (ab52894, Abcam, USA), p-EGFR (ab32430, Abcam, USA) and IRF-1 (ab243895, Abcam, USA). GAPDH (ab8245, Abcam, USA) was used as corresponding controls as indicated.

### 2.7 Mice

Control wild-type (WT) and AECs-specific ITGB4 conditionally knocked out (ITGB4^−/−^) mice were constructed as previously described (24, 25). Briefly, to produce ITGB4^−/−^ mice, doxycycline (Dox; 1% in drinking water for mother mice) was administered to neonatal CCSP–rtTAtg/−/TetO-Cretg/−/ITGB4^fl/fl^ triple transgenic mice for 21 day. All animal studies were proved by No.2020sydw0910 Animal Care and Ethics Committees of Centre South University.

### 2.8 Mice Infection and HDM Administration

Mice were intranasal infected of RSV (1 × 10^6^ PFU in 20μl of virus suspension) at 15 days after birth. As control, mock-infected mice received 20μl of PBS. HDM administration was performed at 6 weeks of age. To construct HDM susceptibility model, Mice were intranasal treated with 75μg HDM in 20μl a week, which is lasted for 3 weeks. Analyses were performed on days 3 after the final HDM administration.

### 2.9 Measurement of Lung Function

Airway resistance was measured using a direct plethysmography (Biosystems XA; Buxco Electronics, U.S.A.), as previously described (26).

### 2.10 Immunofluorescence

Immunofluorescence (IF) was detected and analyzed according to our previous publication (27). The following primary antibodies were used: ITGB4 (ab197772, Abcam, USA), RSV-F (ab24011, Abcam, USA).

### 2.11 Giemsa Staining

BAL fluid was collected as described previously (23). The BALF was centrifuged to remove supernatant, and the precipitate was fixed by pipetting with methanol. The cell suspension is evenly smeared on the glass slide. After air-drying, sample were put into Giemsa working solution A (Solarbio, China) for 30 seconds and transferred into working solution B for 3 minutes before washing with running water. Then, the slide is dried and observed using a light microscope.

### 2.12 Lung Histology Staining

Paraffin-embedded lung sections were stained with hematoxylin and eosin (HE), Glycogen Periodic Acid Schiff (PAS) and Masson, respectively. Immunohistochemical staining was performed with the following antibody: ITGB4 (ab197772, Abcam, USA). Histopathological changes (airways inflammation, mucus secretion and collagen deposition) were scored blindly according to morphological criteria, with reference to previous publications (28).

### 2.13 Flow Cytometry

Single cell suspensions from lungs were prepared as previously described (29) and flow cytometric analysis was operated as previous described (22). Cells were then incubated with corresponding antibodies (eBioscience, San Diego, CA) to detect different cell subpopulations which is shown in **Supplementary Table 2**. Numbers of positive cells were quantified by flow cytometry (FACS Canto flow cytometer, BD Biosciences, San Jose, CA). All flow cytometric data were collected and analyzed with Flow Jo software (version 7.6, Tree Star, Inc).

### 2.14 Data Analysis and Statistics

Statistical analyses were conducted and analyzed by GraphPad Prism 6.0 and SPSS 20.0 statistical software. The independent sample T test was used for the comparison between the two groups, and the one-way ANOVA was used for the comparison of multiple groups. All experiments were repeated more than 3 times, Differences were considered statistically significant for *p <0.05, **p < 0.01 and ***p < 0.001.

## 3 Results

### 3.1 Expression of ITGB4 Decreases Significantly in Nasopharyngeal Samples From Children With RSV Infection

**Figure 1:**
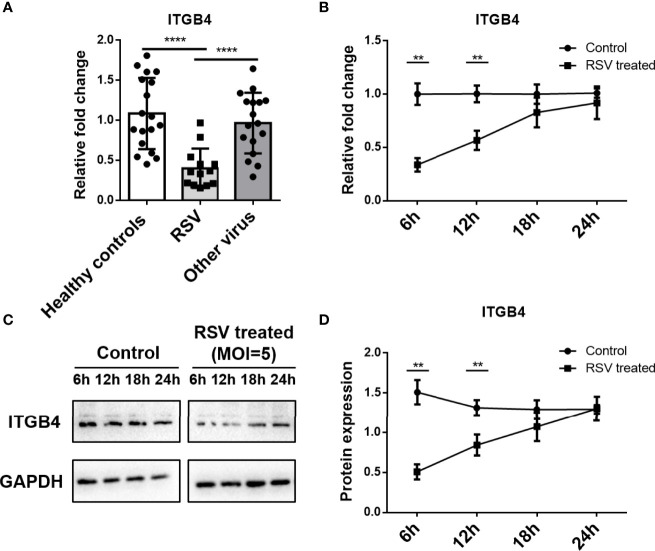
Expression of ITGB4 decreased significantly in nasopharyngeal samples from children with RSV infection. **(A)** The mRNA level of ITGB4 was assessed in nasopharyngeal samples from young children presenting acute respiratory infection with RSV or other respiratory viruses and healthy controls. **(B–D)** The expression of ITGB4 was detected at 6, 12, 18 and 24 hpi in RSV (MOI=5) infected HBE cells. **p < 0.01,****p < 0.0001. hpi: hours post infection.

To address the expression of ITGB4 after RSV infection, nasopharyngeal samples were obtained from young children presenting to hospital with acute respiratory infection and healthy individuals visiting medical center, respectively. Patients with RSV - RNA positive was selected to compare with children infected by other pathogens or healthy controls. Compared with healthy controls or children infected with other respiratory pathogens, the mRNA level of ITGB4 was significantly decreased in the RSV infected children (**Figure 1A**).

### 3.2 RSV Infection Induces to a Transient Decrease of ITGB4 Expression in HBE Cells

**Figure 2:**
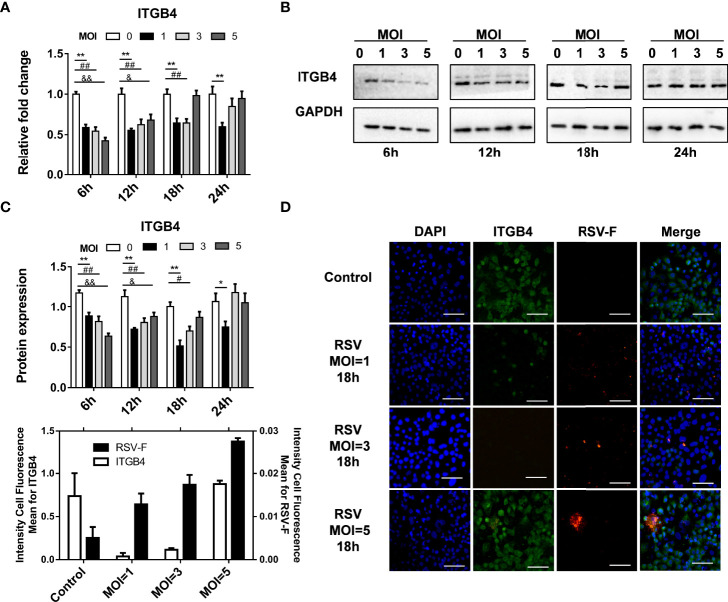
The duration of decreased ITGB4 expression in HBE cells is negatively correlated with expression of RSV related protein. **(A–C)** The level of ITGB4 were detected at 6, 12, 18 and 24 hpi by qPCR and Western blot. **(D)** Representative micrographs of ITGB4 (green) and RSV-F (red) was detected at 18 hpi in HBE cells. Scale bar, 25um. *,#,& p < 0.05; **,##,&& p < 0.01.

RSV-infected HBE cells were used to assess the effect of RSV infection on ITGB4 expression *in vitro*. RSV infection (MOI=5) significantly down-regulated ITGB4 expression at 6 hpi (hours post infection) and 12 hpi. Subsequently, the expression of ITGB4 gradually recovered from 12 hpi to 24 hpi (**Figures 1B–D**). To further explore the regulation mode of ITGB4 expression by RSV infection, HBE cells were infected with RSV in different multiplicity. The mRNA level of ITGB4 decreased from 6 hpi to 12 hpi dramatically. Of note, ITGB4 expression recovered at 18 hpi in MOI 5 group and recovered at 24 hpi in MOI 3 group. Yet, MOI 1 group is remained at low level at 24 hpi (**Figure 2A**). The protein level of ITGB4 was coincide with the mRNA expression (**Figures 2B, C**). Meanwhile, with the increase of MOI, the fluorescent spots number of RSV-F protein increased at 18 hpi (**Figure 2D**). The fluorescence intensity of ITGB4 from the MOI 1 and MOI 3 group were significantly lower whereas no significant difference was detected in MOI 5 group (**Figure 2D**). Correlation analysis showed that the duration of decreased ITGB4 expression was inversely proportional to infection virulence. These results yielded the same conclusion, ITGB4 was down-regulated after RSV infection. Moreover, the duration of decreased ITGB4 expression was associated with the persistent state of RSV infection, which suggested that the differential expression of ITGB4 may controlled by the process of binding and entering cells membranes of RSV, but not the intracellular activities.

### 3.3 RSV Infection Leads to a Transient Decrease of ITGB4 Expression in Neonatal Mice

**Figure 3:**
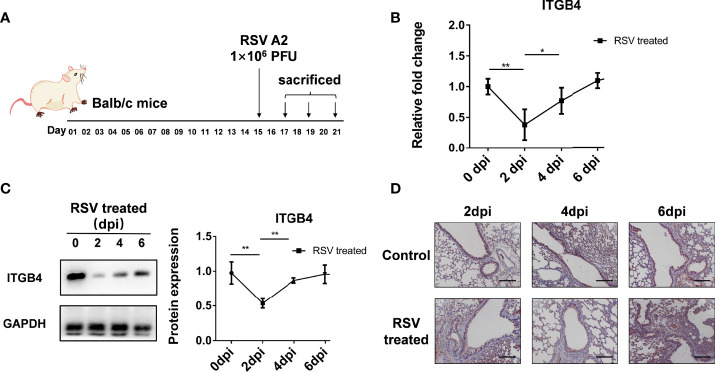
The expression of ITGB4 was decreased transiently in lung tissue of Balb/c mice after RSV infection. **(A)** Protocol for RSV infection in neonatal Balb/c mice. **(B)** The mRNA level of ITGB4 was detected by qRCR. **(C)** The protein level of ITGB4 was assayed by Western blot. **(D)** The *in-situ* expression of ITGB4 was observed by immunohistochemistry. Scale bar, 50 um. *p < 0.05; **p < 0.01.

Neonatal Balb/c mice were also administrated with RSV to detect the influence of RSV infection on ITGB4 expression *in vivo* (**Figure 3A**). The mRNA and protein level of ITGB4 decreased after RSV infection, with lowest point at 2 dpi. Then, ITGB4 expression elevated gradually from 4dpi to 6 dpi (**Figures 3B, C**). Immunohistochemistry further verified that the expression of ITGB4 in airway lumen was significantly reduced at 2 dpi, which was gradually recovered from 4 to 6 dpi, (**Figure 3D**).

### 3.4 ITGB4 Deficiency Induces Higher Viral Burden in RSV-Infected Neonatal Mice

**Figure 4:**
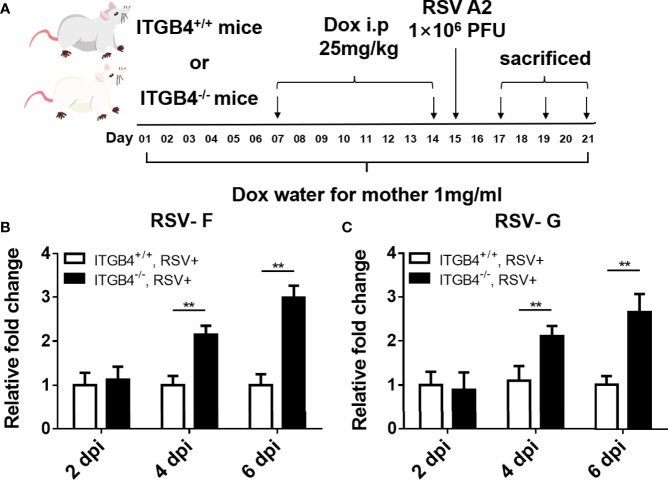
ITGB4 deficiency in airway epithelia led to increased viral load in RSV-infected mice. **(A)** Protocol for administration of RSV in ITGB4 conditional knockout mice. **(B, C)** RSV related genes (RSV-F, RSV-G) in lung tissue were detected at 2, 4 and 6 dpi. **p < 0.01. dpi: days post infection.

ITGB4 conditionally knock out mice were constructed to determine the level of virus clearance in neonatal mice after RSV infection (**Figure 4A**). Using whole lung sample, there is no significant increase in RSV fusion protein (RSV-F) and RSV Glycoprotein G protein (RSV-G) at 2 dpi in either neonatal ITGB4^-/-^ or ITGB4^+/+^ mice. However, compared with ITGB4^+/+^ mice, there was a significant increase of RSV-F and RSV-G expression in ITGB4^-/-^ mice at 4 and 6 dpi following RSV infection (**Figures 4B, C**). These data provide evidence that ITGB4 deficiency in airway epithelia enhanced virus replication and weakened virus clearance in lung after RSV infection.

### 3.5 ITGB4 Deficiency Aggravates Lung Inflammation in RSV-Infected Neonatal Mice

**Figure 5:**
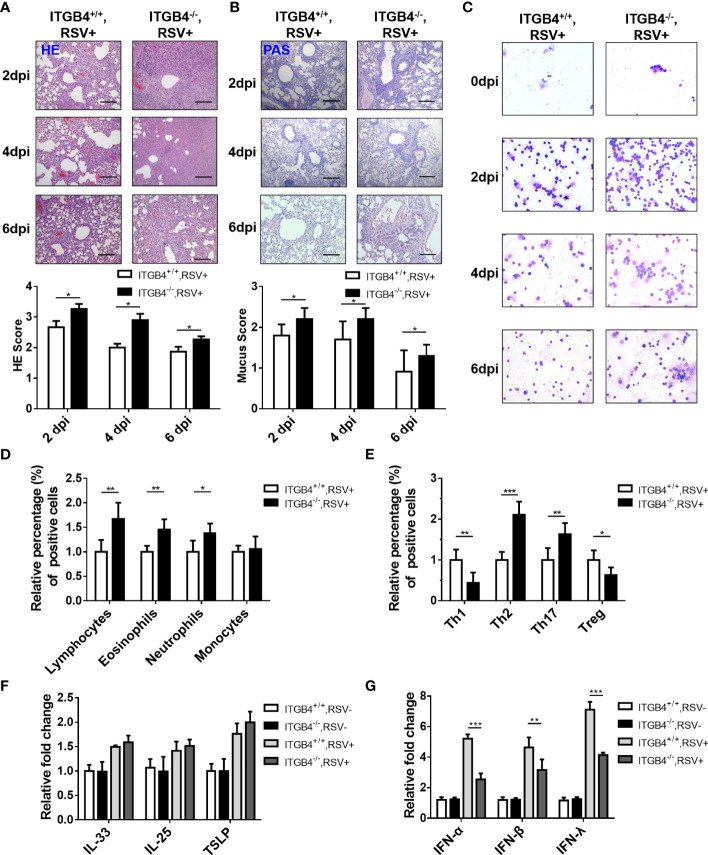
ITGB4 deficiency led to increased Th2 inflammation and decreased IFN production in RSV-infected mice. **(A)** HE staining and HE score. **(B)** PAS staining and mucus score. Scale bar, 50um. **(C)** BALF inflammatory cell was counted (Giemsa×400). Scale bar, 50um. **(D)** Inflammatory cells in lung were counted and classified by flow cytometry. **(E)** T cells subsets were counted and classified by flow cytometry. **(F)** The mRNA level of IL-33, IL-25 and TSLP in mice lung tissue was detected by qPCR. **(G)** The mRNA level of IFN-α, IFN-β and IFN-λ in lung tissue was detected by qPCR. *p < 0.05; **p < 0.01; ***p < 0.001.

Except for increased viral load in lung, compared to ITGB4^+/+^ mice, aggravated lung inflammation in neonatal ITGB4^-/-^ mice was induced after RSV infection. At 2 dpi, lung inflammation and sloughed airway epithelial cells in the airway lumen was detected both in ITGB4^+/+^ mice and ITGB4^-/-^ mice. Of note, the inflammation degree in ITGB4^-/-^ mice were dramatically serious which reached peak at 4 dpi (**Figure 5A**). Similarly, goblet cells in the airway lumen of ITGB4^-/-^ mice were hyperplasia and mucus secretion was increased greatly at 4 and 6 dpi, which was not observed in ITGB4^+/+^ mice (**Figure 5B**). In line with aforementioned findings, the inflammatory infiltrates in BALF of RSV-infected ITGB4^-/-^ mice also augmented significantly compared with control group, and the increased inflammatory cells mainly includes lymphocytes, eosinophils and neutrophils (**Figure 5C, D**). Specifically, the proportion of neutrophils, eosinophils, Th2 and Th17 cells elevated greatly after RSV infection in ITGB4^-/-^ mice and the proportion of Th1 type cells and Treg cells is significantly reduced (**Figure 5E**). These observations reveal that ITGB4 deficiency in airway epithelia exacerbates the RSV-induced lung inflammation.

### 3.6 Involvement of ITGB4 Deficiency on the Decreased Expression of Interferon (IFN) in RSV-Infected Mice

Although IL-33, IL-25 and TSLP are the important epithelia-secreted cytokines that specifically promote Th2-type responses (30), there was no pronounced difference in the expression of IL-33, IL-25, or TSLP in RSV infected ITGB4^-/-^ mice compared with control mice (**Figure 5F**). It is well known that IFNs plays an important role in antiviral responses and airway epithelial cells is the main source of type I and type III IFNs to promote the synthesis of IFN-stimulated genes (ISGs) and limit virus replication. To determine how ITGB4 contributes the IFN_S_, we further evaluated the expression of type I and type III IFNs (IFN-α, IFN-β, IFN-λ) in ITGB4^-/-^ and ITGB4^+/+^ mice after RSV infection. Significant lower levels of IFN-α, IFN-β and IFN-λ was detected in the lung of ITGB4^-/-^ mice after RSV infection, among which IFN-λ was the most significant (**Figure 5G**). Taken together, these findings indicate that ITGB4 in HBE cells critically regulates the expression of IFNs and is associated with the pathogenesis of RSV infection.

### 3.7 ITGB4 Deficiency in Airway Epithelia of Neonatal Mice Increases HDM Sensitivity After Early RSV Infection

**Figure 6:**
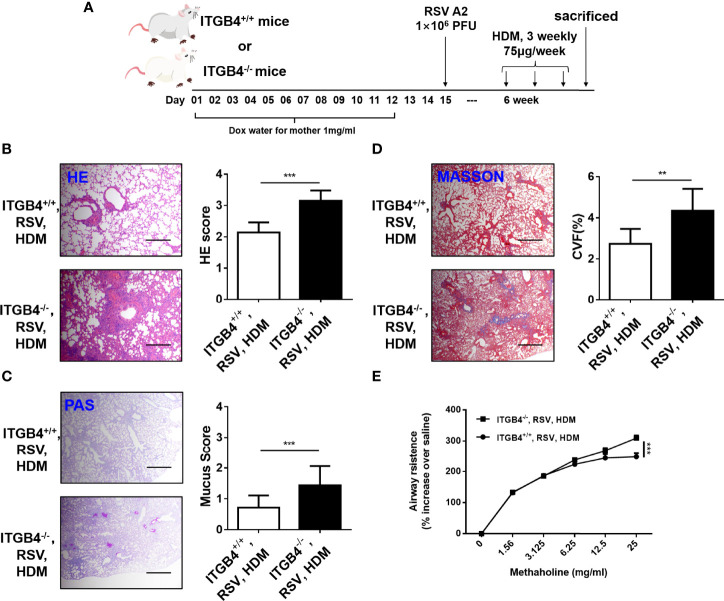
ITGB4 deficiency during RSV infection contribute to HDM sensitivity. **(A)** Protocol for administration of HDM in mice. **(B)** HE staining and HE score. **(C)** PAS staining and PAS score. **(D)** Masson staining and Collagen volume fraction (CVF). Scale bar, 50um. **(E)** AHR was represented as airway resistance in response to methacholine. Data represents the median with range of four mice per group. **p < 0.01, ***p < 0.001 by two-way ANOVA followed by Fisher *post hoc* test.

RSV-induced severe LRTI is an independent risk factor for asthma. As ITGB4 deficiency in airway epithelia of neonatal mice aggravated RSV infection in lung significantly, we further speculated that the enhanced RSV infection caused by ITGB4 deficiency would contribute to the sensitivity of HDM in later life. To avoid experimental asthma caused by secondary injury, conditional knock out of ITGB4 was controlled only during RSV infection. Thus, RSV was administrated on P15 of neonatal period. Mice were exposed to HDM 4 weeks after RSV infection (**Figure 6A**). Consistent with our conjecture, ITGB4 deficiency induced enhanced RSV infection in early life that further increased HDM sensitivity later in later life, which is presented with enhanced lung inflammatory cell infiltration, increased airway mucus secretion, collagen deposition and significant higher AHR after HDM exposure (**Figures 6B–E**). These results demonstrated that early ITGB4 deficiency enhanced the degree of RSV infection, which further affected the severity of asthma pathology after HDM stress in later life. Of note, this effect may not be specific in the situation of ITGB4 deficiency which also applies to all factors that can induced early severe RSV infection. Our synchronous experimental displayed that the HDM-induced asthma phenotype was also exaggerated in early enhanced RSV infection caused by ozone exposure (**Supplementary Figure 1**). Together, these finding confirm the strong connection between early severe RSV infection and development of later asthma.

### 3.8 ITGB4 Regulates the Expression of IRF-1 Through the Phosphorylation of EGFR in RSV-Infected HBE Cells

**Figure 7:**
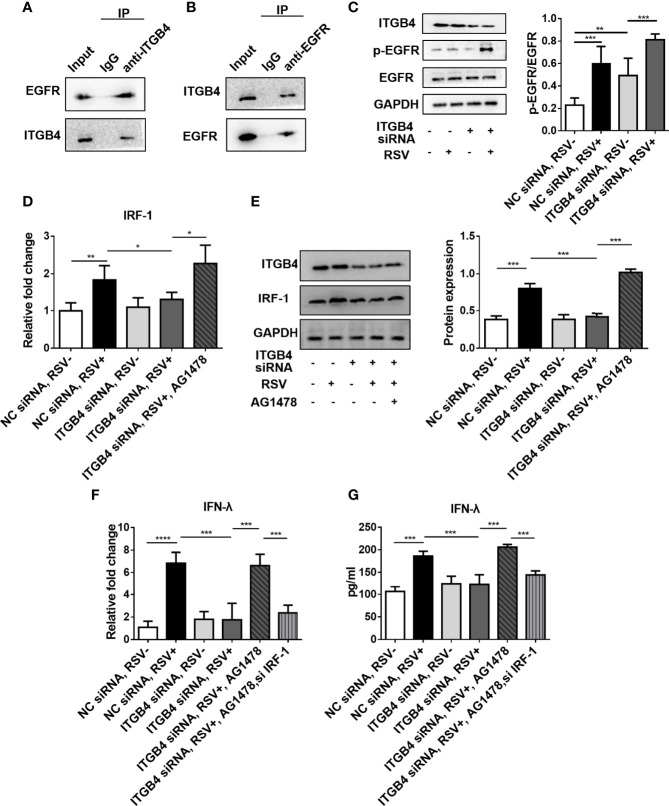
ITGB4 deficiency downregulate IFN-λ expression through EGFR-IRF-1 pathway in RSV-infected HBECs. **(A, B)** The binding of ITGB4 and EGFR were detected by immunoprecipitation. **(C)** The protein level of EGFR and p-EGFR in HBE cells were detected by Western blot. **(D)** The mRNA level of IRF-1 was detected by qRCR. **(E)** The protein level of IRF-1 was detected by Western blot. **(F)** The mRNA level of IFN-λ was detected by qRCR. **(G)** The protein level of IFN-λ was detected by Western blot. *p < 0.05, **p < 0.01,***p < 0.001,****p < 0.0001.

There is a direct interaction between ITGB4 and EGFR (22, 31), and ITGB4 has been shown to regulate EGFR phosphorylation in HBE cells. Intriguingly, considerable literatures confirmed that RSV participates in suppressing antiviral immunity and enhancing airway inflammation through activating EGFR (32). Based on this, we further explore the impact of ITGB4 deficiency on EGFR phosphorylation in RSV-infected HBE cells. In line with aforementioned findings, ITGB4 can direct bind to EGFR (**Figures 7A, B**), and EGFR phosphorylated in airway epithelial cells were inhibited by ITGB4 in HBE cells. RSV infection induced the phosphorylation of EGFR that was further enhanced in ITGB4 silenced airway epithelial cells (**Figure 7C**). Thus, ITGB4 was involved in the regulation of EGFR activation in RSV-infected airway epithelial cells.

As airway epithelial ITGB4 deficiency significantly inhibited the expression of IFN-λ during RSV infection and IRF-1 is an important transcription factor that regulates the expression of IFN-λ. Moreover, IRF-1 can also participate in the pathway of MAV- induced IFN-λ production (33). In this present study, we further found that the expression of IRF-1 was increased after RSV stimulation. However, in ITGB4 silenced HBE cells, the expression of IRF-1 was inhibited after RSV infection which was also validated with EGFR inhibitor (AG1478) (**Figures 7D, E**). In addition, inhibition of IRF-1 decreased the expression of IFN-λ significantly in ITGB4 silenced HBE cells which was restored by AG1478 (**Figures 7F, G**). Collectively, these data indicated that ITGB4 deficiency in airway epithelia enhances EGFR activation and inhibits IRF-1 production after RSV infection, resulting in decreased IFN-λ expression and suppression of antiviral response.

## 4 Discussion

ITGB4 is a down-regulated structural adhesion molecule in the airway epithelia of asthma patients which is strongly associated with asthma susceptibility (12). It has also been verified that ITGB4 deficiency in airway epithelia leads to enhanced Th2 airway inflammatory response and exaggerated AHR after HDM stress. RSV infection in airway epithelial cells is the main cause of bronchiolitis in children, which is closely associated with wheezing and increased susceptibility of asthma in later childhood. Of note, the intracellular fragment of ITGB4 contain two pairs of fibronectin-like domains, which strongly indicated the potential effect of ITGB4 on the pathological response of RSV infection. In this present study, we further revealed that the expression of epithelial ITGB4 in RSV-infected mice was down-regulated at the early stage of RSV infection, which gradually returned to initial level in the late stage of RSV infection. As mice are not the natural hosts of RSV and not highly susceptible to RSV, we deduced that the restoration of ITGB4 expression in RSV-infected epithelial cells is more relevant to the effective elimination of RSV. Impressively, the *in vitro* RSV-infected cellular model observed a transient low expression of ITGB4. Moreover, the recovery speed of ITGB4 expression was negatively correlated with intracellular virulence. The symptoms after infection determines infection outcomes during RSV infection in airway epithelia. These phenomena suggest that the differential expression of ITGB4 in airway epithelia occur in the invading process of RSV, which are related to the signal transduction process of interaction between RSV and epithelial cells membranes. To date, considerable researchers have found that the surface proteins and non-structural proteins of RSV have immunomodulatory properties, which can interfere the antiviral response and immune defense by adopting different strategies (29, 34–36). Thus, the decreased expression of ITGB4 in the early stage of RSV infection may be not only a critical evasion mechanism but also a host immune response in RSV-infected airway epithelial. As a critical adhesion molecule of airway epithelial cells, ITGB4 participates in the conduction of multiple signal pathways, including wound repair, immune regulation and antiviral defense, etc. (13–15). The decreased expression of ITGB4 in the early stage of RSV infection may induce the obstacle of the airway barrier function, delay the subsequent antiviral response and even causes abnormal immune response in respiratory tract after RSV infection. Therefore, RSV attack in ITGB4-deficient airway epithelia would exacerbate the severity of RSV infection. In this regard, our study makes sense for understanding the heterogeneity of RSV infection, which also support the gene-environment interactions that underpin the occurrence disease (37).

To further explore the contribution of ITGB4 on RSV infection *in vivo*, a neonatal RSV-infected mice model was constructed using our well-established ITGB4 conditional knockout mouse. In line with aforementioned findings, the virus clearance ability was weakened in RSV-infected neonatal ITGB4^-/-^ mice. Moreover, the magnitude of inflammation was aggravated and the mucus secretion in the bronchial lumen was remarkable increased. Specially, the infiltration of neutrophils and eosinophils in ITGB4^-/-^ mice was increased, which is accompanied by imbalance of Th1 and Th2 cells. These immunopathologic changes justified that ITGB4 deficiency in airway epithelia induce the different immune response in RSV-infected mice which is Th2 dominance. More than that, ITGB4 deficiency in airway epithelia of neonatal mice increases antigen sensitivity after early RSV infection. Experiments in mice demonstrated that early infection of RSV in ITGB4 deficient mice caused not only exacerbated RSV-induced pathological symptoms, but also enhanced lung inflammatory cell infiltration, airway mucus secretion and collagen deposition after HDM exposure in later life, which suggests a critical role for ITGB4 deletion in virus-induced asthma. More than that, the major cytokines that secreted from airway epithelia during RSV infection were also altered. Notably, the secretion of antiviral IFN I and IFN III were significantly inhibited in ITGB4 deficient mice after RSV infection, while no obvious differences were detected among inflammatory cytokines IL-33, IL-25 and TSLP. It suggested that ITGB4 deficiency in airway epithelia inhibits antiviral immunity mainly through the IFN pathway, rather than directly promoting Th2-type responses. In our *in vivo* RSV-infected mice model, the inhibitory effect of ITGB4 deficiency on type III IFN (IFN-λ) is also the most prominent. Although IFNs I can directly inhibit virus replication in respiratory and gastrointestinal epithelia, IFN III is the most critical interferon for virus clearance (38). In addition, IFN-λ has been shown to mediate adaptive immunity. Previous studies have demonstrated that IFN-λ regulates the differentiation of T cells in the absence of IFN-γ, which can also decrease the expression of IL-4 and IL-13 (39, 40). Meanwhile, the transcription of type III IFN is dependent on NF-κB pathway, while the IRF pathway dominates the expression of type I IFN. Moreover, IRF-1 can also specifically promotes the expression of type III IFN (41, 42). Although ITGB4 deficiency in airway epithelia induced a high pathogenicity of RSV infection both in *in vitro* and *in vivo*, the intrinsic mechanisms are still not fully understood.

Our previous work has verified that ITGB4 can bind to EGFR and reduce the binding efficiency of EGFR and other ligands in airway epithelial cells (14, 43). Recent studies further shown that virus-induced epithelial EGFR activation can inhibit the antiviral innate immune response (33, 38, 44). EGFR is activated in RSV-infected AECs which also enhanced airway inflammation and increased mucin production (45, 46). The use of EGFR inhibitors can significantly increase the secretion of IFN-λ in the RSV-infected airway epithelia which is IRF-1 dependent (32). On this basis, this study further confirmed that ITGB4 deficiency increased EGFR phosphorylation and decreased IRF-1 expression in RSV-infected airway epithelial cells which can be restored by EGFR inhibitors. Thus, ITGB4 deficiency weakened the anti-virus ability of airway epithelia through the EGFR/IRF-1/IFN-λ pathway in RSV-infected airway epithelial cells. There is also similar established regulatory axis of IRF-1/IFN-λ signaling in the hepatic responses to Klebsiella pneumoniae infection (47). This was an unexpected finding that the difference in IFN was detected, but not in proinflammatory cytokine. Thus, the mechanism of enhanced virus load after RSV infection in ITGB4 deficient airway epithelia still requires further investigation.

There are still some limitations in this study. Firstly, the nasopharyngeal aspirate samples were obtained from the patients at the time of admission. However, it was hard to record the duration of the RSV infection when samples were collected at the time of admission. It is also uncertain whether the duration of RSV infection is consistent with that of other respiratory viruses. Secondly, the expressions of some cell surface molecules differ between cells from emerged monolayer and differentiated airway epithelium (48). Compared with 2D cell cultured technique, HBE cultured in Air-Liquid Interface can better mimic the *in vivo* properties of airway epithelial cells, which is a more ideal model to study the effects of infectious agents on epithelial cells. We will consider constructing the HBE cultured in Air-Liquid Interface to better mimic the *in vivo* properties of airway epithelia in our future work.

In summary, RSV infection in early life is heterogeneous and illustrating the determinant of RSV-infected phenotype is crucial to avoid severe infection and subsequent asthma susceptibility. This study confirmed that ITGB4 deficiency in airway epithelial induced weakened lung antiviral response and aggravated lung inflammatory during RSV infection through decreased secretion of IFN-λ. Understanding the molecular mechanism of heterogeneous characteristics in RSV-infected airway epithelia may provide new useful strategy for the prevention and treatment of RSV infection.

## Data Availability Statement

The raw data supporting the conclusions of this article will be made available by the authors, without undue reservation.

## Ethics Statement

The studies involving human participants were reviewed and approved by No.2020KT-52. Written informed consent to participate in this study was provided by the participants’ legal guardian/next of kin. The animal study was reviewed and approved by No.2020sydw0910.

## Author Contributions

XD and CL conceived and designed this study. XD, LY, YeY, YuY, KZ, XW, and LW carried out the experiments. MY, LQ, WL, YX, XPQ, HL, and XQQ contributed to the interpretation of the results. CL and XD wrote the paper. All authors provided critical feedback and helped shape the research, analysis and manuscript. All authors contributed to the article and approved the submitted version.

## Funding

This work was funded by grants #82070034, #81970033, #31900424 from the NSFC; grants #2019JJ50760, #2020JJ4776, #2020JJ4688, #2021JJ30898, #2021JJ31090 from the Hunan Natural Science Foundation; grant #20K142 from open Foundation of Hunan College Innovation Program, grant #2020SK5370 from the innovation guidance project of clinical medical technology in Hunan province, and the grant for Open Sharing Fund for the Lager-scale Instruments and Equipment’s of Central South University.

## Conflict of Interest

The authors declare that the research was conducted in the absence of any commercial or financial relationships that could be construed as a potential conflict of interest.

## Publisher’s Note

All claims expressed in this article are solely those of the authors and do not necessarily represent those of their affiliated organizations, or those of the publisher, the editors and the reviewers. Any product that may be evaluated in this article, or claim that may be made by its manufacturer, is not guaranteed or endorsed by the publisher.

## Supplementary Material

Supplementary Table 1 Primer sequence of genes for qPCR.

Supplementary Table 2 Cell marker antibodies for flow cytometry analysis.

Supplementary Figure 1 Ozone attack (O_3_) during RSV infection contribute to HDM sensitivity. **(A)** Protocol for ozone attack, RSV infection and administration of HDM in mice. **(B)** HE staining and HE score. **(C)** PAS staining and PAS score. **(D)** Masson staining and Collagen volume fraction (CVF). Scale bar, 50um. **(E)** AHR was represented as airway resistance in response to methacholine. Data represent the median with range of four mice per group. **p<0.01 by two-way ANOVA followed by Fisher *post hoc* test.

## Abbreviations

RSV, Respiratory syncytial virus; AECs, Airway epithelial cells; ITGB4, Integrin β4; IFN, Interferon; HDM, House Dust Mite; MOI, Multiplicity of Infection.
