# Supplementary material for: ITGB4 Deficiency in Airway Epithelium Aggravates RSV Infection and Increases HDM Sensitivity
Source: Front Immunol. 2022 Jul 25;13:912095. doi: 10.3389/fimmu.2022.912095 (PMC9357881; doi:10.3389/fimmu.2022.912095)
Supplement: Supplementary Table 1 — Primer sequence of genes for qPCR. [file Table_1.docx]

**Supplementary Table 1.** Primer sequence of genes for qPCR.

| Gene |  | Sequences |
| --- | --- | --- |
| ITGB4 (human) | Forward | CACCTCCGTCTCCTCCCAC |
|  | Reverse | GTTGGGGATGTTGAGCCGAT |
| β-actin (human) | Forward | TTGCAGCTCCTTCGTTGCC |
|  | Reverse | GACCCATTCCCACCATCACA |
| RSV-F | Forward | CTGTGATAGARTTCCAACAAAAGAACA |
|  | Reverse | AGTTACACCTGCATTAACACTAAATTCC |
| RSV-G | Forward | CAACGCCAAAACAAACCACC |
|  | Reverse | GGCTTGGTGGTGGTTTTCTT |
| ITGB4 (mouse) | Forward | CCGCTTCCAAGTCCAACTCA |
|  | Reverse | TGAGACTCCTGTCCGTTTCA |
| β-actin (mouse) | Forward | TTGCAGCTCCTTCGTTGCC |
|  | Reverse | GACCCATTCCCACCATCACA |
| IL-33 (mouse) | Forward | TCCAACTCCAAGATTTCCCCG |
|  | Reverse | CATGCAGTAGACATGGCAGAA |
| IL-25 (mouse) | Forward | CAGCAAAGAGCAAGAACCCC |
|  | Reverse | ACCCGATTCAAGTCCCTGTC |
| TSLP (mouse) | Forward | ACGGATGGGGCTAACTTACAA |
|  | Reverse | AGTCCTCGATTTGCTCGAACT |
| IFN-α (mouse) | Forward | CATTCTGCAATGACCTCCAC |
|  | Reverse | TCAGGGGAAATTCCTGCAC‐3 |
| IFN-β (mouse) | Forward | ATGACCAACAAGTGTCTCCTCC |
|  | Reverse | GGAATCCAAGCAAGTTGTAGCTC |
| IFN-λ (mouse) | Forward | CGAAGAACAAGAAGCGGAACG |
|  | Reverse | AAGGTTGAACAGTCCGATGCG |
| IFN-λ (human) | Forward | GGGAACCTGTGTCTGAGAACGT |
|  | Reverse | GAGTAGGGCTCAGCGCATAAATA |
| IRF-1 (human) | Forward | CTCTGAAGCTACAACAGATGAGG |
|  | Reverse | CTGTAGACTCAGCCCAATATCCC |
