# Supplementary material for: ITGB4 Deficiency in Airway Epithelium Aggravates RSV Infection and Increases HDM Sensitivity
Source: Front Immunol. 2022 Jul 25;13:912095. doi: 10.3389/fimmu.2022.912095 (PMC9357881; doi:10.3389/fimmu.2022.912095)
Supplement: Supplementary Table 2 — Cell marker antibodies for flow cytometry analysis. [file Table_2.docx]

**Supplementary Table 2.** Cell marker antibodies for flow cytometry analysis.

| Cell types | markers |
| --- | --- |
| Neutrophils, monocytes | CD45 (FITC), CD115 (Percp/CY5.5), LY-6C (PE), LY-6G (APC), CD11b (PE/CY7) |
| Eosinophils, B cells | CD45 (FITC), Siglec-F (PE), CD19 (APC), I-A/I-E （Percp/CY5.5） |
| Total T cells, Th1, Th2, Th17 | CD45 (FITC), CD3(BV510), CD4(Percp/CY5.5), IFN-γ (APC), IL-4 (PE), IL-17 (PE-CY7) |
| Treg | CD45 (FITC), CD25 (APC), FOXP3(PE) |
