# Supplementary figures and images for: ITGB4 Deficiency in Airway Epithelium Aggravates RSV Infection and Increases HDM Sensitivity
Source: Front Immunol. 2022 Jul 25;13:912095. doi: 10.3389/fimmu.2022.912095 (PMC9357881; doi:10.3389/fimmu.2022.912095)

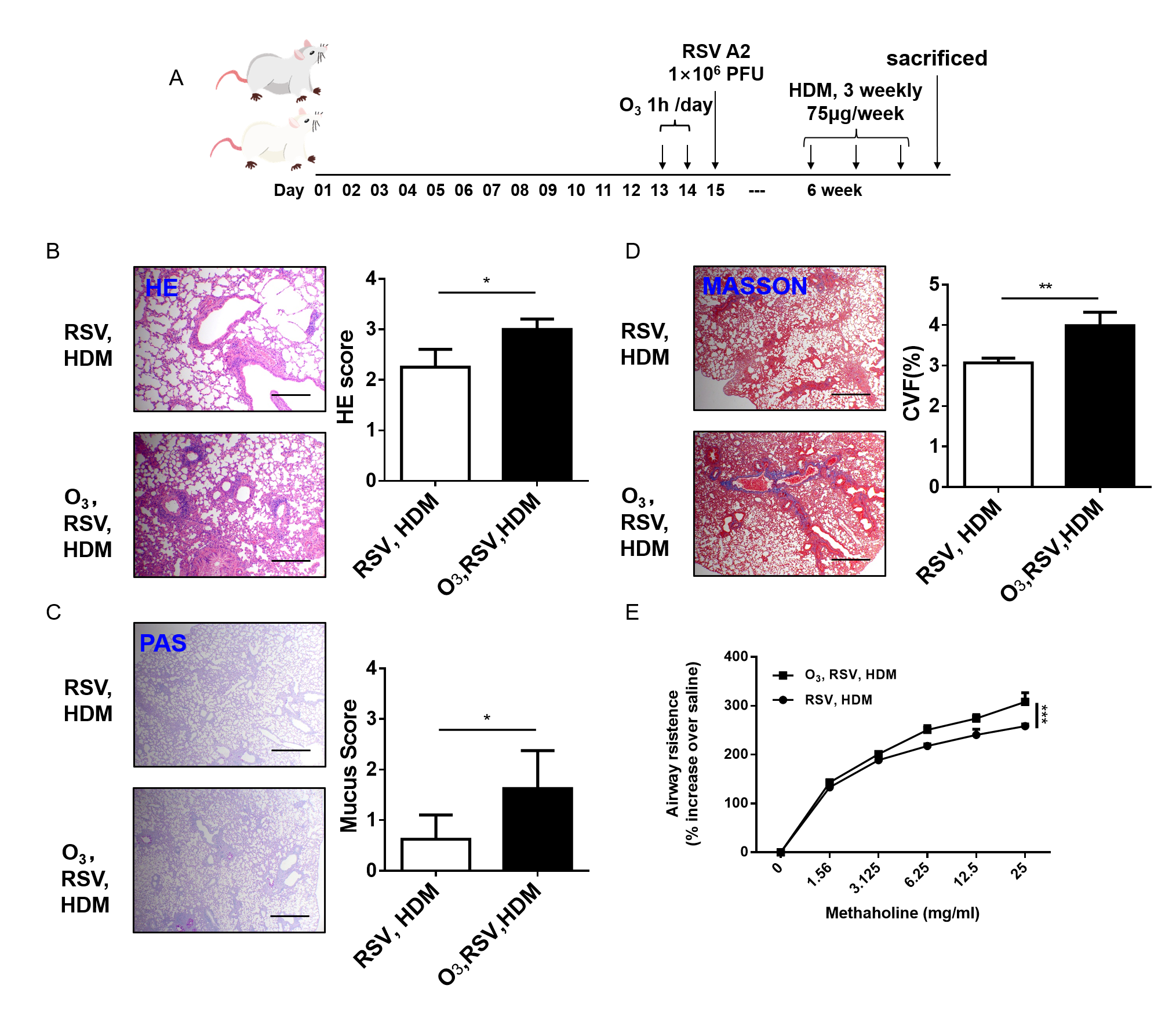

Supplement: Supplementary Figure 1 — Ozone attack (O3) during RSV infection contribute to HDM sensitivity. (A) Protocol for ozone attack, RSV infection and administration of HDM in mice. (B) HE staining and HE score. (C) PAS staining and PAS score. (D) Masson staining and Collagen volume fraction (CVF). Scale bar, 50um. (E) AHR was represented as airway resistance in response to methacholine. Data represent the median with range of four mice per group. **p<0.01 by two-way ANOVA followed by Fisher post hoc test. [file Image_1.tif]
